# Narrative Review of Potential Non-Surgical Treatments for BCG-Unresponsive Non-Muscle Invasive Bladder Cancer

**DOI:** 10.3390/jcm15103830

**Published:** 2026-05-15

**Authors:** Sung Han Kim, Haarika Gudlavalleti, Seth P. Lerner

**Affiliations:** Scott Department of Urology, Dan L Duncan Cancer Center, Baylor College of Medicine, Houston, TX 77030, USA; sunghan.kim@bcm.edu (S.H.K.);

**Keywords:** bladder cancer, non-muscle-invasive, radical cystectomy, recurrence, Bacillus Calmette–Guérin

## Abstract

Intravesical Bacillus Calmette–Guérin (BCG) is a recommended therapy approach for non-muscle invasive bladder cancer (NMIBC) to prevent disease progression and eliminate tumor cells. Nevertheless, some patients have shown resistance to BCG therapy, exhibiting disease recurrence and progression to MIBC, thereby necessitating radical cystectomy as the best-recommended treatment option. However, radical cystectomy greatly affects postoperative morbidity and quality of life, rendering many patients hesitant or surgically unsuitable for this invasive therapy. The concept of bladder preservation has emerged as an alternative, employing a diverse array of therapeutic agents and interventions to improve therapeutic efficacy to retain the bladder for as long as possible. In this review, we explore various established therapeutic agents and ongoing clinical trials involving new potential agents and interventions. This study was undertaken to determine the advantage of each agent and their combined therapeutic regimens in improving prognostic efficacy, offering an alternative to radical cystectomy for BCG-unresponsive NMIBC.

## 1. Introduction

Non-muscle-invasive bladder cancer (NMIBC), confined to the urothelium (Ta, Tis) or lamina propria (T1), accounts for approximately two-thirds of all bladder cancer cases. Intravesical Bacillus Calmette–Guérin (BCG) remains the standard adjuvant therapy, achieving a 6-month complete response rate of up to 84% in carcinoma in situ (CIS) and 2- and 5-year recurrence-free survival (RFS) rates of 82% and 62%, respectively, in high-risk patients with adequate induction and maintenance BCG [1,2,3,4,5]. Nevertheless, many patients relapse after adequate BCG and enter the BCG-unresponsive (unBCG) disease state, defined in consensus with the US Food and Drug Administration (FDA) as the persistence or recurrence of CIS (with or without Ta/T1) within 12 months of adequate BCG, high-grade Ta/T1 recurrence within 6 months, or T1 HG at the first post-induction evaluation; for these high-risk patients, guidelines recommend radical cystectomy (RC) as the standard of care, with durable complete remission rates of approximately 60–70% [6,7,8,9].

Because RC carries substantial morbidity and affects quality of life, the FDA has created a single-arm registration pathway in unBCG CIS with and without papillary disease, and multiple data sets suggest that RC can be safely delayed in carefully selected patients: a retrospective series of 278 BCG-refractory cases showed no adverse effects on disease specific mortality when RC was deferred up to one year after transurethral resection of bladder tumor (TURBT), a prospective cohort of 1529 BCG-treated patients with NMIBC found progression to be uncommon within 6 months with a median time to progression of approximately 24 months for T1 HG, and in KEYNOTE 057, none of the 102 unBCG CIS patients progressed over a median 15.8-month follow-up [10,11,12]. Consequently, this integrative review focuses on currently validated bladder-sparing therapies with diverse mechanisms, including intravesical agents, immunotherapies, and emerging gene-based strategies for the management of unBCG NMIBC, aiming to support delayed cystectomy and durable bladder preservation, which is oncologically appropriate [13].

## 2. Methods

We employed a comprehensive search strategy using the keywords “bladder cancer,” “BCG,” “non-muscle-invasive,” “unresponsive,” and “treatment” to systematically identify the relevant literature published between 2021 and 2026. A systematic search was conducted across multiple public databases, including PubMed (n = 77), Embase (n = 37), the Cochrane Library (n = 53), ClinicalTrials.gov (n = 17), and major conference proceedings (ASCO GU, ESMO, AUA, EAU; n = 1228). After removing duplicates and excluding non-core journals, conference abstracts without follow-up data, and studies that did not report outcomes in unresponsive BCG (unBCG) NMIBC, a total of 87 articles were ultimately selected from a systematic search and their findings were synthesized narratively based on evidence-based clinical studies published within the past 5 years. These 87 publications were categorized as representing either potential practice-changing evidence (phase 3 clinical trial) or promising investigational therapies (phase 1 and 2 and other real-world series), according to an independent review of full-text articles, abstracts, and clinical trials involving unBCG NMIBC cohorts, with or without control groups, conducted by the investigators (Supplementary Table S1). No formal risk-of-bias assessment was performed, as this was a narrative review rather than a quantitative meta-analysis.

## 3. Main Body

Over the past decade, advances in molecular science have led to the development of new agents and interventional strategies for treating unBCG NMIBC [14]. Currently, FDA-approved options include intravesical valrubicin (only for CIS), nadofaragene firadenovec, and nogapendekin alfa inbakicept combined with BCG, as well as systemic pembrolizumab for patients with unBCG CIS with or without Ta/T1 high-grade disease; sequential intravesical gemcitabine–docetaxel has become a widely adopted community standard despite the lack of level 1 evidence from prospective randomized trials [14] (Table 1).

These therapies have expanded the potential for delayed RC and bladder preservation and are complemented by emerging modalities, such as novel intravesical chemotherapies, photodynamic therapy, immunotherapy, targeted agents, and gene-based approaches, all of which may further support organ-sparing strategies in appropriately selected patients with unBCG NMIBC.

### 3.1. Intravesical Chemotherapy

#### 3.1.1. Valrubicin

Valrubicin (Valstar^TM^), a semisynthetic analog of the anthracycline doxorubicin, was approved by the FDA in 1988 for patients with unBCG NMIBC with CIS of the bladder who were not candidates for immediate cystectomy [13]. In a study by Steinberg et al. involving 90 patients with recurrent CIS after initial BCG treatment, weekly instillations of valrubicin (800 mg for six weeks) resulted in a 21% complete response (CR) rate and a 7.8% disease-free survival (DFS) rate lasting up to 30 months [15]. In the A9303 phase II/III open-label trial, Valrubicin achieved a 41% 3-month CR and an overall 18% CR, with a 16.5-month CR median duration of response in patients with BCG-refractory disease or BCG intolerance [16].

#### 3.1.2. Gemcitabine

Gemcitabine, a deoxycytidine nucleoside analog, is one of the most widely used intravesical chemotherapeutic agents for the treatment of NMIBC. A single postoperative instillation of intravesical gemcitabine within 24 h of resection has been used to reduce recurrence rates and has become the standard of care for patients with low- and intermediate-risk NMIBC [7]. It inhibits DNA synthesis and can be used as a single agent or in combination with other chemotherapy drugs [17]. In a study by Hurle et al. [17], gemcitabine administered to 36 patients as an induction regimen, followed by maintenance therapy in responders, resulted in DFS and recurrence-free survival (RFS) rates of 44%, 32%, 80%, and 70% at 12 and 24 months, respectively. The cancer-specific survival (CSS) and overall survival (OS) at 24 months were 81% and 78% [17]. In the phase II SWOG 0353 study by Skinner et al. [18], 58 patients treated with intravesical induction of gemcitabine alone once weekly for 6 weeks achieved CR rates of 47% at 3 months and 21% at 24 months, with a median RFS of 6.1 months. Thirty-six percent of patients underwent RC [18]. Despite some favorable outcomes, a standardized regimen remains elusive because of variability across studies owing to considerable heterogeneity in study designs, with differential purposes such as either induction or maintenance therapy. Therefore, no definitive conclusions can be drawn regarding intravesical chemotherapy after BCG administration.

#### 3.1.3. Combination Therapies

Combination therapies involving gemcitabine have also been administered to patients with NMIBC, following BCG therapy. These combinations include gemcitabine and docetaxel [19,20], cabazitaxel and cisplatin [21], oral everolimus [22], and mitomycin C (MMC) [23,24]. Steinberg et al. showed that Gem/Doce achieved durable oncological outcomes, with RFS rates of 54% at 12 months and 34% at 24 months in 45 patients, whereas 10 patients ultimately underwent cystectomy [19]. Chevuru et al. reported gemcitabine–docetaxel RFS rates of 57%, 44%, and 24% at 12, 24, and 60 months, respectively [20]. Docetaxel alone demonstrated CR rates of 56–59% [25].

A phase I trial involving 18 participants with unBCG NMIBC evaluated the efficacy of triple intravesical therapy with cabazitaxel, gemcitabine, and cisplatin [21]. The results indicated good tolerability and potential efficacy of the regimen. In a triplet study by De Castro et al., a combination of gemcitabine with cabazitaxel and cisplatin yielded better results, with an 89% CR rate, a median RFS of 27 months, and RC-free survival rates of 81% and 94% at 1 and 2 years, respectively [21]. In a study by Dalbagni et al., the combination of oral everolimus with gemcitabine yielded 3-, 6-, and 12-month RFS rates of 58%, 27%, and 20%, respectively [22]. Moreover, Cockerill and Lightfoot showed that the combination with MMC yielded a CR rate and 1- and 2-year RFS rates of 68%, 48%, and 38%, respectively [23,24].

### 3.2. Targeted Therapy

Mutations in fibroblast growth factor receptor 3 (FGFR3) serve as prognostic biomarkers of BC [26,27]. The Hahn and Hoosier Cancer Research Network Trial (HCRN 12-157) evaluated dovitinib for treatment-resistant, molecularly enriched NMIBC, including unBCG NMIBC with increased phosphorylated FGFR3 expression. The study revealed a 19-month DFS and an overall 6-month CR rate of 8% (33% of patients with FGFR3 mutations), whereas no response was observed in 11 patients without FGFR3 mutations [27]. In addition, the THOR-2 study (NCT04172675) highlighted erdafitinib, an oral selective pan-FGFR tyrosine kinase inhibitor, in patients with unBCG NMIBC and CIS exhibiting FGFR alterations with or without concurrent papillary disease [28,29].

### 3.3. Immunotherapy

Intravesical administration of monoclonal antibodies, particularly humanized programmed cell death protein 1 (PD-1) agents known as immune-checkpoint inhibitors (ICIs), has several potential advantages over systemic administration (Table 2). The notable benefit of ICIs is their direct effect on the tumor environment while minimizing toxicity, making them increasingly crucial in BC therapy [30,31]. Trials with various ICIs, including pembrolizumab, atezolizumab, nivolumab, durvalumab, avelumab, tislelizumab, and vicinium, have been administered to patients who experience recurrence after BCG therapy.

#### 3.3.1. Pembrolizumab

Pembrolizumab, a PD-1 ICI, is currently approved for treating patients with high-risk unBCG NMIBC harboring CIS with or without papillary tumors and who are ineligible for or have chosen not to undergo surgery, based on the results of the phase II KEYNOTE-057 study [32,33]. This trial included 96 patients who received intravenous pembrolizumab, and CR was observed in 41% of patients (n = 39), with a median 16.2-month CR duration of response and a CR of 46% at 12 months [33]. Another ongoing phase III KEYNOTE-676 trial (NCT03711032), which evaluates the combination of BCG and pembrolizumab versus BCG monotherapy in 550 patients with persistent or recurrent high-risk NMIBC after BCG induction, was launched with the primary endpoints of CR (CIS cohort) and event-free survival [34].

#### 3.3.2. Atezolizumab

Atezolizumab was evaluated in a SWOG phase II single-arm trial involving 172 patients with unBCG high-risk NMIBC (NCT02844816) [35]. Of the 74 patients with CIS, 27% achieved CR at 6 months, with a median response duration of 17 months, and 56% of those who achieved CR experienced a durable response lasting at least 12 months. Of the 129 eligible patients, 12 experienced progressions to MIBC or metastatic disease.

#### 3.3.3. Tislelizumab

Tislelizumab (BGB-A317) is a humanized IgG4 anti-PD-1 ICI specifically designed to minimize binding to Fc gamma receptors in macrophages. It has also been evaluated in patients with unBCG and NMIBC in a previous study, which demonstrated the clinical advantages of tislelizumab in locally advanced or metastatic PD-L1-positive urothelial carcinoma [36]. Li et al. evaluated the efficacy and safety of tislelizumab in combination with radiotherapy as a bladder-sparing strategy in 14 patients with unBCG and found encouragingly positive results, with a bladder preservation rate of 100% at 24 months and a remarkable OS rate at the same time point [37].

#### 3.3.4. Nivolumab, Durvalumab, and Avelumab

In the CheckMate 9UT trial involving unBCG NMIBC with CIS and with or without papillary disease (NCT03519256), nivolumab monotherapy, nivolumab plus BMS-986205 (oral indoleamine 2,3-dioxygenase 1 inhibitor), nivolumab plus BCG, and nivolumab plus BCG and BMS-986205 were evaluated to assess the proportion of CIS patients achieving a CR rate and the duration of CR, with a time frame of up to 5 years [37,38,39]. In a phase II single-arm trial in patients with BCG failure (NCT03759496), durvalumab was administered through weekly intravesical instillations over a total of 6 weeks, with the primary endpoint being the HG progression-free survival (PFS) rate at 30 days and 6 months after instillation and therapy, respectively [40]. Another durvalumab study involving a multi-arm, multi-stage phase I/II study (NCT03317158) investigated the safety and efficacy of durvalumab monotherapy (cohort 1), durvalumab in combination with BCG (cohort 2a), and external beam radiation therapy (cohort 2b) in patients with unBCG NMIBC [41]. The PREVERT trial (NCT03950362) focused on IV avelumab administered for 6 months to evaluate the synergistic efficacy of avelumab in combination with bladder radiotherapy [37,42,43].

#### 3.3.5. Nogapendekin Alfa Inbakicept (NAI)

Nogapendekin alfa inbakicept (NAI, N 803 or ALT 803, Anktiva) is an IL 15-based immunostimulatory complex that binds a dimeric human IL 15Rα/IgG1 Fc and activates NK cells, CD8^+^ T cells, and effector/memory T cells, showing synergistic anti-tumor activity when combined with BCG [44,45]. In the phase 2/3 QUILT 3.032 trial (NCT02773849), N 803 plus BCG achieved robust and durable efficacy in high-risk unBCG NMIBC: in the CIS with and without papillary cohort, the anytime CR rate was 71% (58/82) with a median duration of response of 26.6 months, 53% of initial complete responders remained in CR at 24 months, approximately 89–90% avoided cystectomy, and disease-specific survival at 24 months was 100%, and in the high-grade Ta/T1 papillary-only cohort, the 12-month DFS rate was 55% with a median DFS of 19.3 months; most treatment emergent adverse events were grades 1–2 and grade 3 immune-related events were rare [46], leading to FDA approval in April 2024 for unBCG NMIBC with CIS with or without papillary tumors and positioning N 803 plus BCG as a new reference standard for patients who were ineligible for or declined RC [44,47].

In parallel, an ongoing phase Ib/II multicenter study (NCT03022825) has evaluated N 803 in combination with intravesical gemcitabine in patients with unBCG NMIBC who are unwilling or ineligible for RC [48,49]. This trial included an initial dose escalation phase Ib to define safety and the recommended dose, followed by an expansion phase II in which both agents were administered intravesically, with the primary aim of characterizing the CR rate, duration of response, and event-free survival in this high-risk population, and exploring N 803-based combinations as additional bladder-sparing options beyond N 803 plus BCG.

#### 3.3.6. Recombinant T-Cell Receptor-IL-2 Fusion Protein, ALT-801

Another recombinant humanized T-cell receptor (TCR)-IL-2 fusion protein, ALT-801, enhances the cytotoxic immune responses of NK and T cells against p53-expressing tumor cells (NCT01625260) [40,50,51]. In this phase I trial, Sonpavde et al. [52] found that intravenous infusion of ALT-801 and gemcitabine demonstrated exploratory favorable durable clinical activity in patients with unBCG high-risk NMIBC, defined as HG Ta, T1, or CIS; a tumor size greater than 4 cm; or multifocal tumors. CR was observed in three of six patients, and in two of them, it was durable (≥18 months) [51,52,53]. This trial demonstrated not only feasibility and exploratory durable clinical activity in a small cohort of unBCG high-risk NMIBC patients, establishing systemic T-cell receptor-targeted immunotherapy as a novel bladder-preserving strategy with potential for precision application in p53-mutated tumors, but also provided the first proof of concept that systemic immunotherapy could overcome the limitations of intravesical therapy, enabling niche positioning in treatment-refractory cases.

### 3.4. Gene Therapy

Gene therapy targets the genetic instability of tumors at both the chromosomal and nucleotide levels, considering the rapidly evolving tumor microenvironment, genotypic and phenotypic alterations that facilitate tumor invasion, and the development of tumor escape mechanisms [54].

#### 3.4.1. Nadofaragene Firadenovec (rAd-IFNα/Syn3, ALDASTRIN)

Nadofaragene firadenovec (rAd-IFNα/Syn3), also known as ALDASTRIN, is the most recent FDA-approved agent for unBCG NMIBC. Nadofaragene firadenovec is a non-replicating recombinant adenovirus vector-based gene therapy agent that delivers a copy of the human interferon-α-2b gene into the urothelial cell wall [55,56], whereas syndecan-3 (Syn3) is a polyamide surfactant that enhances viral transduction of the urothelium [57].

In a phase III multicenter single-arm trial in 151 patients with unBCG NMIBC and CIS with or without high-grade Ta/T1, Boorjian et al. reported that 55 of 103 CIS patients (53.4%) achieved CR within 3 months of the first dose and that this was maintained at 12 months in 25 of those 55 responders (45.5%), with a single intravesical administration sufficient to generate measurable urinary IFN α and overall CR rates with nadofaragene firadenovec ranging from 29% to 60% at 3 months and 29% to 35% at 12 months [58,59]. The 60-month OS was 80% (71.0, 86.0) including 76% (64.6–84.5) for the CIS cohort and 86% (70.9–93.5) in the Ta/T1 cohort. Only five patients (four with CIS and one with Ta/T1) experienced clinical progression to MIBC [60]. Complementing these pivotal data, a real-world multicenter Mayo Clinic cohort of 46 patients with unBCG NMIBC treated with nadofaragene firadenovec showed that among 24 evaluable CIS with or without papillary cases, 79% achieved a 3-month CR; 68% (13/19) of responders remained in CR at a median 9.3-month follow-up; and in 19 papillary-only patients, high-grade RFS at 3, 6, and 12 months was 68%, 53%, and 26%, respectively. With a 13.9-month median follow-up, cystectomy-free survival and OS were 93% and 95%, and toxicity was mainly grade 1–2 bladder spasms (61%) and failure to retain instillation (33%) with only four grade 3 events and no grade 4–5 events, while a pooled phase 2/3 safety review confirmed a consistent, favorable intravesical safety profile for nadofaragene firadenovec across studies [61,62].

#### 3.4.2. Diphtheria Toxin A (BC-819)

Another non-viral plasmid gene therapy, BC 819 (inodiftagene vixteplasmid), uses the conjugated H9 promoter and a polyethyleneimine plasmid (jetPEI™) with a selective gene encoding the expression of diphtheria toxin A for enhanced cell transfection and blocking protein synthesis, leading to tumor cell death [63,64,65,66]. In a preliminary study involving 18 patients with unBCG NMIBC, 22% exhibited confirmed expression of H19, resulting in the complete disappearance of tumor markers without the appearance of a new tumor [63]. Furthermore, 5 of the 18 patients demonstrated a DFS of >35 weeks. However, studies focused specifically on high-risk unBCG NMIBC have demonstrated limited efficacy, leading to the discontinuation of treatment [64,65,66].

NCT03719300 (CODEx study), an open-label, single-arm, multicenter phase II trial in high-risk unBCG NMIBC (mainly CIS with or without papillary disease), was a key study for intravesical BC-819 at 20 mg in 50 mL once weekly for 10 weeks as induction, followed by maintenance every 3 weeks for up to 84 weeks in responders, with complete response at 12 weeks (disappearance of CIS by cystoscopy, cytology, and biopsy) as the primary endpoint [64]. In the CODEx trial, only 3 of 17 evaluable patients (17.6%) achieved a 12-week CR, failing to meet the predefined efficacy threshold; therefore, a futility analysis led to early termination of the trial despite acceptable low-grade local and systemic adverse events [65,66].

#### 3.4.3. Detalimogene Voraplasmid (EG-70)

Detalimogene voraplasmid (EG-70) is a non-viral intravesical vector plasmid gene that encodes IL-12 and retinoic-acid-inducible gene I to trigger adaptive and innate immune responses, respectively [67]. When administered intravesically, plasmids are delivered directly to the mucosal tissue of the bladder to ensure a localized immune response. A recent phase 1/2 LEGEND trial (NCT04752722) involving the intravesical administration of EG-70 for high-risk unBCG NMIBC with CIS with and without papillary disease reported exploratory favorable early efficacy and a favorable safety profile with an overall CR rate of approximately 68–73% in evaluable CIS patients, supporting the selection of 0.8 mg/mL on a four instillation schedule as the recommended phase 2 dose and triggering the initiation of the pivotal phase 2 cohort [68,69,70]. The ongoing phase 2 cohort showed that among 21 efficacy-evaluable unBCG CIS patients with and without Ta/T1, there was an overall CR rate of 71% (15/21), with 3- and 6-month CR rates of 67% (14/21) and 47% (8/17), respectively, and a 6-month CR estimate of 51%, whereas safety in 42 treated patients remained largely limited to grade 1–2 urinary adverse events without new safety signals, leading to continued enrollment of both CIS- and papillary-only cohorts and positioning EG-70 as a competitive bladder-preserving option in this setting [69,70]. This LEGEND trial (NCT04752722) addresses a critical gap in unBCG NMIBC management, achieving 67–73% 3-month CR rates that substantially exceed FDA benchmarks (≥25%) and positioning detalimogene voraplasmid as one of the first-line bladder-preserving alternatives to RC, with a favorable safety profile dominated by manageable urinary adverse events.

### 3.5. Emerging Therapies (Vaccine Therapy and Other Antigen–Antibody Therapy)

#### 3.5.1. PANVAC

Serial vaccine trials have targeted BCG-mediated diseases, including unBCG NMIBC [71]. One such trial involved a recombinant poxviral vaccine named PANVAC. This vaccine activates CD4+ and CD8+ antigen-specific responses to tumor antigens, carcinoembryonic antigens, and mucin-1 [72,73]. In a phase 2 trial (NCT02015104), the efficacy of the combination of BCG with PANVAC versus BCG monotherapy in patients with high-grade unBCG NMIBC is currently under evaluation, with DFS as the primary outcome [74,75].

#### 3.5.2. Cretostimogene Grenadenorepvec (CG0070)

Cretostimogene grenadenorepvec, also known as CG0070, is a selective oncolytic adenovirus engineered to express GM CSF and preferentially replicate in bladder cancer cells via a defective retinoblastoma pathway, thereby inducing immunogenic cell death and making it an attractive intravesical agent for patients with unBCG NMIBC [76,77,78]. In a phase II single-arm multicenter trial (NCT02365818) led by Packiam et al., 45 patients with unBCG NMIBC who underwent RC and received intravesical CG0070 achieved a 12-month durable CR rate of 29%; a 6-month CR rate of 47% in the overall cohort and 50% in those with pure CIS, with benefits particularly notable in the pure CIS subgroup; and toxicity largely limited to grade 1–2 local adverse events [79]. This trial established CG0070 as a viable bladder-preserving treatment option, representing the first successful case leveraging selective replication in retinoblastoma-pathway-deficient tumors and positioning it as a reasonable first-line alternative for patients refusing RC. More recently, the phase II CORE1 trial evaluated CG0070 in combination with pembrolizumab, reporting a 3-month CR rate of 87.5%, with 68% of evaluable responders maintaining a durable CR at 1 year. Most adverse events were mild and transient that the combination of CG0070 with PD-1 inhibition demonstrated synergistic efficacy, with an initial CR rate of 87.5% representing the highest level among bladder-preserving treatments and showing better response rates than pembrolizumab monotherapy (41%) [78]. These two phase II trials, both as monotherapy and in combination with pembrolizumab, demonstrate compelling efficacies in unBCG NMIBC, establishing oncolytic virotherapy as a leading bladder-preserving strategy with synergistic potential when combined with ICIs.

#### 3.5.3. Vesigenurtacel-L (HS-410)

A transdermal vaccine called Vesigenurtacel-L (HS-410) is also being tested in a phase I/II study (NCT02010203) involving BCG-naïve or unBCG NMIBC. This vaccine was derived from an allogeneic BC cell line transfected with gp96-Ig to stimulate CD-8+ cytotoxic cell production [80,81]. However, vesigenurtacel-L development was discontinued because a phase 1/2 trial (NCT02010203) did not demonstrate a clear, clinically meaningful improvement over BCG alone, despite showing acceptable safety and evidence of immune activation.

#### 3.5.4. Enfortumab Vedotin (EV)

Enfortumab vedotin (EV) is an antibody–drug conjugate directed against nectin-4. A phase 1, open-label, multicenter study led by the study group of Kamat et al. is currently underway to evaluate the safety, tolerability, and anti-tumor activity of intravesical EV administered weekly for six induction instillations followed by monthly maintenance, with planned dose levels of 125, 250, 500, and 750 mg in patients with unBCG NMIBC (CIS with or without papillary disease) who were ineligible for or declined RC (NCT05014139) [82]. The interim analysis reported that three of the four patients treated at 125 mg achieved CR, while the fourth discontinued treatment because of persistent disease; no dose limiting toxicities or grade ≥3 treatment-related adverse events were observed.

### 3.6. Device-Assisted Therapies (Hyperthermia and Photodynamic Therapy)

Hyperthermia and photodynamic therapy (PDT) are advanced therapeutic modalities that enhance the effects of intravesical therapy in patients with unBCG NMIBC.

#### 3.6.1. Hyperthermia (HIVEC^®^)

The hyperthermic intravesical chemotherapy (HIVEC^®^) technique, a form of chemohyperthermia, enhances the efficacy of intravesical MMC by using an intravesical heating system to raise the bladder urothelial temperature to approximately 44–48 °C, which improves drug penetration into the urothelium, exerts direct cytotoxic effects on tumor cells, and increases apoptosis. In the large prospective multicenter HIVEC E study, 172 of 1028 patients with NMIBC were classified as unBCG, and the 12- and 24-month RFS rates were 78.1% and 57.4%, respectively; at 24 months, RFS was 43.6% for CIS with or without papillary disease and 64.5% for papillary-only disease, with severe adverse events occurring in only 2% of patients [83]. Nativ et al. evaluated intravesical MMC delivered with RF-induced hyperthermia in patients with recurrent NMIBC after prior BCG and observed that this combined thermo-chemotherapy produced a meaningful complete response and RFS in a substantial subset of patients with high-risk unBCG, with toxicity mainly limited to manageable local urinary symptoms [84]. Similarly, de Jong et al. studied HIVEC^®^ (recirculating hyperthermic intravesical MMC) in 55 patients with unBCG NMIBC and reported a 3-month complete response rate of 70% in those with concomitant CIS, a median DFS of 17.7 months, a 1-year cumulative recurrence/progression incidence of 53%, and low rates of severe adverse events, supporting hyperthermic chemotherapy as a salvage bladder-preserving strategy in this setting [85].

Although hyperthermia-based intravesical therapy has shown favorable outcomes in these trials and series, it has not yet been regarded as the standard first-line treatment for NMIBC. The current research focuses on refining optimal treatment protocols, better defining patient selection, and clarifying the long-term safety and oncological benefits of hyperthermia, both as a standalone intravesical approach and in combination with other modalities for NMIBC.

#### 3.6.2. Photodynamic Therapy (PDT)

Photodynamic therapy (PDT) involves leveraging the interaction between absorbed light and a retained photosensitizing agent to destroy tumor tissue [86]. The efficacy of PDT with padachlorin was reported in a study involving 34 patients with high-grade NMIBC who were refractory or intolerant to BCG. This study demonstrated RFS rates of 90.9%, 64.4%, and 60.1% at 12, 24, and 30 months, respectively [87]. In a phase 1 trial involving patients with unBCG NMIBC, PDT resulted in CR in 45% of patients at 3 months [88]. PDT appears to be more effective in patients with CIS and unBCG NMIBC than in those presenting with papillary recurrence.

## 4. Discussion

In unBCG NMIBC, RC remains the standard recommended treatment. However, with the introduction of new agents and combinational therapeutic approaches, there is growing interest in prolonging bladder preservation by reducing PFS and RFS events. In this review, we focused on potential non-surgical bladder-preserving treatment options based on clinical trials. Larger multicenter studies have provided either potential practice-changing evidence or promising investigational therapies. While some agents remain in phase 1/2 exploratory stages, others have demonstrated efficacy in phase 3 trials, with an increasing impact on real-world clinical practice in unBCG NMIBC.

In NMIBC, BCG remains a cornerstone of guideline-based therapy, and its efficacy in BCG-naïve disease is influenced by several factors, including BCG strain, re-TURBT, and maintenance therapy. Del Giudice et al. reported that BCG TICE showed a superior 5-year recurrence-free survival compared with RIVM in 852 propensity-score-matched patients, particularly when guideline-recommended re-TUR and maintenance schedules were followed; however, no significant differences were observed in PFS or cancer-specific survival (CSS) [89].

After the development of unBCG NMIBC, the AUA BC guidelines recommend prioritizing enrollment in clinical trials whenever feasible, particularly those evaluating investigational agents with promising activity, such as cretostimogene grenadenorepvec and detalimogene voraplasmid. When trial participation is not possible, current bladder-sparing options include nadofaragene firadenovec, pembrolizumab, nogapendekin alfa inbakicept (N-803), and sequential intravesical gemcitabine/docetaxel (Table 3).

Among the FDA-approved agents, pembrolizumab, nadofaragene firadenovec, and nogapendekin alfa inbakicept differ meaningfully in mechanism of action, durability of response, toxicity, and practical accessibility (Table 4). N-803 plus BCG appears to provide the most durable responses, particularly in patients with CIS [42,49], whereas nadofaragene firadenovec offers a favorable balance of efficacy and tolerability, along with the convenience of infrequent intravesical dosing [58,59,60]. Pembrolizumab remains an important bladder-sparing option for selected patients; however, responses are generally less durable and treatment is associated with systemic immune-related adverse events [32,33].

From a real-world perspective, nadofaragene firadenovec is often the most practical intravesical option because of its limited systemic toxicity and relatively simple administration schedule [58,59,60]. Pembrolizumab may be more widely available in oncology practices; however, its higher cost and systemic toxicity profile can limit its use. In contrast, the combination of N-803 plus BCG may be highly effective, but its use depends on BCG availability and local implementation, which may restrict accessibility in some settings [44,45].

Current evidence supports a stratified approach to treatment selection for unBCG NMIBC, prioritizing RC for fit patients with high-risk features while reserving bladder-preserving therapies for those with papillary-only disease, CIS ± papillary tumors, or significant comorbidities (Table 5) [3,7,31]. For patients with CIS ± papillary tumors, first-line therapy favors N-803 + BCG (grade 2A evidence; 71% CR rate), followed by pembrolizumab (phase 2, grade 2B; 41% CR) or nadofaragene firadenovec (Adstiladrin; grade 1B; 53% CR), reflecting FDA approvals and superior level 1B/2A evidence over investigational options [44,58,59,60]. In papillary-only cases (which carry a lower risk of progression), gemcitabine/docetaxel (grade 2B) demonstrates exceptional efficacy (100% 3-month CR), with HIVEC-MMC (grade 2B; 78% 12-month RFS) as second-line, enabling high organ preservation rates supported by multicenter real-world data [19,20,83,84,85]. For high-comorbidity patients (ECOG ≥ 2, RC ineligible), valrubicin (grade 1B; 18–21% CR) or single-agent gemcitabine (grade 2B; 47% CR) offers tolerable intravesical alternatives with minimal systemic toxicity, aligning with guideline recommendations for frail populations where phase 1B/2B evidence prioritizes safety over aggressive RC [3,7,31]). RC candidates (T1 + CIS + LVI+, fit; grade 1A) remain the gold standard (80% 5-year OS), but patient refusal shifts to the above hierarchy, ensuring evidence-based personalization that balances progression risk, organ preservation, and comorbidity burden [7].

This narrative review did not systematically assess publication bias or comprehensively evaluate all therapeutic agents. Instead, it primarily focused on non-surgical treatment options for unBCG NMIBC, with particular emphasis on phase 2 and 3 studies, while phase 1 data were included only when relevant preliminary findings were available. Although many agents remain under active investigation, the lack of head-to-head trials, baseline heterogeneity among enrolled cohorts, and the fact that several phase III trials are still pending mean that not all agents are suitable for every patient with unBCG NMIBC, and the limitations and toxicity profiles of each treatment must be carefully considered. In addition, personalized risk stratification may further refine treatment selection beyond conventional pathology. For example, a retrospective analysis of 228 patients undergoing RC showed that the pre-surgical systemic inflammation response index (SIRI), using a cutoff of 1.71 × 10^9^/L, was independently associated with advanced pT stage, loco-regional extension, and poorer relapse-free survival, while the decision curve analysis demonstrated added clinical net benefit for RFS, CSS, and OS models [90].

Future progress will likely depend on the successful development of new therapies as well as rational combinations with existing treatments to maximize bladder preservation, survival outcomes, and quality of life while avoiding RC whenever possible. These findings suggest that low-cost biomarkers such as SIRI may enhance prognostication and help individualize treatment decisions in patients with bladder cancer. Further studies integrating novel therapies with established modalities, including surgery and, in selected settings, radiotherapy, may help define more effective and patient-tailored strategies for this challenging disease.

## 5. Conclusions

A substantial proportion of patients with unBCG NMIBC require alternative RC, particularly those who are ineligible or unwilling to undergo surgery. In recent years, various innovative therapeutic approaches have emerged, demonstrating the potential to control intravesical disease while preserving the bladder and maintaining quality of life. In carefully selected patients, these bladder-sparing modalities can provide short-term oncologic outcomes that approach those with RC. However, randomized comparative studies are needed to establish whether these therapies provide comparable short- and long-term outcomes, particularly given the heterogeneity of patient populations and differences in oncologic endpoints among patients who are ineligible for RC.

Multiple intravesical regimens have been shown to reduce tumor recurrence, and the development of novel immunotherapeutic agents—such as checkpoint inhibitors and cytokine-based immunotherapy—continues to expand the treatment landscape. Gene- and viral-vector-based therapies are also showing early promise, although additional studies are needed to confirm their long-term safety and efficacy. Because not all patients derive equal benefit from these approaches, individualized treatment planning, multidisciplinary evaluation, and close surveillance remain essential to optimize both disease control and quality of life. Ongoing clinical and translational research will be critical to establish durable evidence and define the most effective bladder-preserving strategies for this challenging patient population.

## Figures and Tables

**Table 1:** Treatment options for unBCG NMIBC.

Treatment	Administration Route	FDA Approval (Approval Year)	For BCG-Unresponsive NMIBC	Status/Notes
Valrubicin (Valstar™)	Intravesical	Yes (2000)	Yes (CIS)	A9303 trial
Gemcitabine	Intravesical	No (off-label)	Yes (off-label)	SWOG 0353
Gemcitabine intravesical system (Inlexzo™)	Intravesical	Yes (2025)	Yes (CIS ± papillary)	SunRISe-1 cohort 2 (NCT04640623)
Gemcitabine + Docetaxel	Intravesical	No (off-label/investigational)	Yes	Multiple studies
Cabazitaxel + Gemcitabine + Cisplatin	Intravesical	No (Phase 2)	Yes	NCT02202772 (phase 2)
Pembrolizumab	Intravenous	Yes (2024)	Yes (CIS)	KEYNOTE-057
Atezolizumab	Intravenous	No (Phase 2)	Yes	SWOG S1605
Tislelizumab	Intravenous	No (Phase 2)	Yes	ChiCTR2000035275
Nivolumab	Intravenous	No (Phase 2)	Yes	NCT03519256
Durvalumab	Intravenous	No (Phase 2)	Yes	NCT03759496
Avelumab	Intravenous	No (Phase 2)	Yes	NCT03317158
Nogapendekin alfa inbakicept (NAI)	Intravesical	Yes (2024)	Yes	Anktiva^®^
ALT-801	Intravenous	No (Phase 1)	Yes	TCR-IL2 fusion
Nadofaragene firadenovec	Intravesical	Yes (2022)	Yes (CIS)	Adstiladrin^®^
Diphtheria toxin A (BC-819)	Intravesical	No (Phase 2)	Yes	CODEx study
Detalimogene voraplasmid (EG-70)	Intravesical	No (Phase 2)	Yes	LEGEND trial
PANVAC	Intramuscular	No (Phase 2)	Yes	Vaccine
Cretostimogene grenadenorepvec (CG0070)	Intravesical	No (Phase 3)	Yes	CORE1 trial
Vesigenurtacel-L (HS-410)	Intramuscular	No (Phase 2)	Yes	Vaccine
Enfortumab vedotin (EV)	Intravenous	No (Phase 2)	Yes	ADC (EV-301)
Hyperthermia (HIVEC^®^)	Device-assisted intravesical	No (investigational)	Yes	HIVEC-HR trial (EudraCT 2016-001186-85)
Photodynamic therapy (PDT)	Device-assisted intravesical	No (investigational)	Yes	NCT03945162

**Table 2:** Summary of ongoing immune agent trials.

Trial Name (NCT ID)	Phase and Patient Population	Interventions	Sample Size	Primary Endpoint(s)	Key Efficacy Findings	Safety Profile
CheckMate 9UT (NCT03519256)	2; unBCG NMIBC with CIS ± papillary tumors	Nivolumab alone; Nivolumab + BMS-986205; Nivolumab + BCG; Nivolumab + BCG + BMS-986205	436	CR rate and duration in CIS patients	Ongoing	Ongoing
Durvalumab Monotherapy (NCT03759496)	2; Patients with unBCG NMIBC	Intravesical Durvalumab	17	6-month CR rate and high-grade PFS rate at 30 days and 6 months	12% CR at 6 months (2/17)	41% immune-related AEs; 1 grade 3 AE
ADAPT-BLADDER (NCT03317158)	1/2; unBCG NMIBC	Durvalumab alone; Durvalumab + BCG; Durvalumab + EBRT	28	Safety and efficacy: RP2D; CR at 6 months,	85% CR at 3mo (Durvalumab + BCG); 73% CR at 12mo	1 grade 3 AE (autoimmune hepatitis)
PREVERT (NCT03950362)	2; unBCG NMIBC	Intravenous Avelumab + EBRT (60–66 Gy)	67	Synergic efficacy of combination therapy: 1-year RFS	ongoing (single arm)	ongoing

NMIBC, non-muscle-invasive bladder cancer; CIS, carcinoma in situ; BCG, intravesical instillation of Bacillus Calmette–Guérin; unBCG, BCG-unresponsive; CR, complete remission rate; PFS, progression-free survival; CR, complete remission rate; RFS, recurrence-free survival; RP2D, recommended phase 2 dose; EBRT, external beam radiation therapy; AE, adverse event.

**Table 3:** Bladder-preserving treatment options from the American Urological Association 2024 [7,11].

Treatment Agents	Patient Cohort	CR Rate (Number of Patients)	12-Month RFS (DOR, Months)	Cystectomy-Free Rate	Major Trial
Nadofaragene firadenovec (Adstiladrin^®^)	CIS ± Ta/T1	51% (50/98)	13% for CIS and 33% for Ta/T1 (57 months)	80% (1yr)	CS-003 (Phase 3)
Pembrolizumab	CIS ± Ta/T1	41% (39/96)	41–46% (16.2 months)	72% (1yr)	KEYNOTE-057 (Phase 2)
Nogapendekin alfa (N-803, Anktiva^®^) + BCG	CIS ± Ta/T1	71% (58/81)	59% (26.5 months)	85% (1yr)	QUILT-3.032 (Phase 2/3)
Valrubicin	CIS only	18% 6-month CR (14/80)	16.4% (11.3 months)	65% (1yr)	A9303 (Phase 2/3)
Gemcitabine/Docetaxel	unBCG NMIBC	66% treatment success rate (30/45)	54% (13.0 months)	78% (1yr)	Steinberg RL, et al. [19]

HR, high-risk; BCG, intravesical instillation of Bacillus Calmette–Guérin; unBCG, BCG-unresponsive; NMIBC, non-muscle invasive bladder cancer; CIS, carcinoma in situ; CR, complete remission rate; RFS, recurrence-free survival; DOR, median duration of response.

**Table 4:** Comparison of Pembrolizumab, Nadofaragene Firadenovec, and Nogapendekin Alfa Inbakicept (N-803).

Item	Pembrolizumab [32,33,34]	Nadofaragene Firadenovec [58,59,60,61,62]	N-803 (Nogapendekin Alfa Inbakicept) + BCG [44,45,46,47]
Key trial/phase	KEYNOTE-057, phase 2	CS-003, phase 3	QUILT-3.032, phase 2/3
Patient population	BCG-unresponsive high-risk NMIBC, especially CIS with or without papillary disease; often used when cystectomy is not feasible or is declined	BCG-unresponsive high-risk NMIBC, especially CIS with or without papillary tumors	BCG-unresponsive NMIBC, including CIS and papillary cohorts
Durability of response	CR rate 41% at 3 months; 46% of responders maintained response for at least 12 months; median CR duration 16.2 months	Durable bladder preservation signal with long-term follow-up; 5-year data showed about half of CIS patients-maintained bladder preservation, with median cystectomy-free survival of 58 months	Strong durability signal: CIS cohort CR 71% with median duration of 26.6 months, and long-term data suggested sustained bladder-sparing benefit
Toxicity profile	Systemic immune-related toxicity; grade 3/4 adverse events 13%, serious treatment-related adverse events 8%	Mostly local urinary adverse events; generally, grade 1–2, with very low rates of severe toxicity and no major new long-term safety signal	Mostly manageable toxicity; mostly grade 1–2 adverse events, with relatively few grade 3 immune-related events; local BCG-related symptoms are common
Patient selection	Best for patients who want bladder-sparing therapy and can tolerate systemic immunotherapy	Best for patients who want an intravesical option with limited systemic toxicity	Best for patients who can continue BCG-based intravesical treatment and want a bladder-sparing approach

BCG, intravesical instillation of Bacillus Calmette–Guérin; NMIBC, non-muscle invasive bladder cancer; CIS, carcinoma in situ; CR, complete remission rate.

**Table 5:** Guide for Therapeutic Clinical Decision-Making in BCG-Unresponsive NMIBC.

Patient Profile	1st Line (Trial Name, Evidence of Grade)	CR Rate	12mo RFS/CFS	2nd Line (Evidence of Grade)	CR Rate	12mo RFS/CFS	3rd Line (Evidence of Grade)	Notes
CIS ± Papillary (High progression risk)	N-803 + BCG (QUILT-3.032, 2A)	71% (58/82)	59% 90% CFS	Pembrolizumab (KEYNOTE-057, 2B)	41% (39/96)	46%/72%	Adstiladrin (CS-003, 1B)	FDA-approved RC refusal priority
Papillary-Only (Lower progression risk)	GemDoce (IRB J2020, 2B)	100% (25/25)	92% CFS	HIVEC-MMC (HIVEC-E, 2B)	Not applicable	78%	CG0070 (2B)	Best bladder preservation
High Comorbidity (ECOG ≥ 2, RC ineligible)	Valrubicin (A9303, 1B)	18–21% (14/80)	16%	Gemcitabine (SWOG 0353, 2B)	47% (27/58)	92%/92%	Clinical trial (3)	Minimal systemic toxicity
RC Candidates (T1 + CIS + LVI+, fit)	Radical Cystectomy (1A)	80% 5yr OS						Gold standard

BCG, intravesical instillation of Bacillus Calmette–Guérin; GemDoce, intravesical instillation of Gemcitabine and Docetaxel; MMC, intravesical instillation of mitomycin-C; NMIBC, non-muscle invasive bladder cancer; RC, radical cystectomy; CR, complete remission rate; RFS, recurrence-free survival; CFS, cystectomy-free survival; OS, overall survival.

## Data Availability

All data shown are publicly available.

## Supplementary Materials

The following supporting information can be downloaded at: https://www.mdpi.com/article/10.3390/jcm15103830/s1, Table S1: Level of evidence.

## Abbreviations

The following abbreviations are used in this manuscript:

AE	Adverse events
BC	Bladder cancer
BCG	Bacillus Calmette–Guérin
CIS	Carcinoma in situ
CR	Complete response rate
CSS	Cancer-specific survival
DFS	Disease-free survival
EBRT	External beam radiation therapy
EpCAM	Epithelial cell adhesion molecule
FDA	Food and drug administration
FGFR3	Fibroblast growth factor receptor 3
ICI	Immune-checkpoint inhibitor
MMC	Mitomycin C
NMIBC	Non-muscle invasive bladder cancer
OS	Overall survival
PD-1	Programmed cell death protein 1
PDT	Photodynamic therapy
PFS	Progression-free survival
RC	Radical cystectomy
rAd-IFNα/Syn3	Nadofaragene firadenovec
RFS	Recurrence-free survival
RP2D	Recommended phase 2 dose
SIRI	Systemic inflammation response index
TURBT	Transurethral resection of bladder tumor
unBCG	BCG unresponsive
