# Supplementary material for: Narrative Review of Potential Non-Surgical Treatments for BCG-Unresponsive Non-Muscle Invasive Bladder Cancer
Source: J Clin Med. 2026 May 15;15(10):3830. doi: 10.3390/jcm15103830 (PMC13208041; doi:10.3390/jcm15103830)
Supplement: Supplementary file 1 [file jcm-15-03830-s001.zip › jcm-4209472-supplementary.pdf]

Supplementary Table S1. Level of evidence

**Study Type**	**Strength**
**Practice-changing evidence**	
Phase III RCT	Highest
Single-arm Phase III	High
**Promising investigational therapies**	
Phase II RCT	Moderate-High
Single-arm Phase II	Moderate
Phase I / Real-world	Low
Case series	Very Low
